# Autoantibodies Against Type I Interferons and Death from H7N7 Avian Flu

**DOI:** 10.1056/NEJMc2608446

**Published:** 2026-11-12

**Authors:** Qian Zhang, Ron A. M. Fouchier, Marcela Moncada-Velez, Adam Meijer, Yingzi Cui, Anne Puel, Paul Bastard, Aurelie Cobat, Shen-Ying Zhang, Ruyue Chen, Ryan A. Langlois, Adolfo García-Sastre, Jean-Laurent Casanova

**Affiliations:** 1 The University of Texas Southwestern Medical Center, Dallas; 2 Erasmus, Rotterdam, the Netherlands; 3 Rockefeller University, New York; 4 Rijksinstituut voor Volksgezondheid en Milieu, Utrecht, the Netherlands; 5 Institut Imagine, Paris; 6 University of Minnesota, Minneapolis; 7 Icahn School of Medicine at Mount Sinai, New York

## To the Editor:

Viruses emerging from zoonotic reservoirs are a major threat to humankind. Since the 1918 H1N1 “Spanish flu” pandemic, four influenza A viruses, three coronaviruses, three hemorrhagic fever viruses, two retroviruses, and two paramyxoviruses have crossed the species barrier and achieved massive human-to-human transmission, with devastating consequences. Since the 1997 Hong Kong outbreak of H5N1 influenza, human infections with 20 other avian influenza viruses have been documented, albeit without sustained human-to-human transmission. In addition to the >1,000 patients killed by H5N1, H7N9, and H5N6 worldwide, four other strains (H7N7, H3N8, H9N2, H10N8) have occasionally led to isolated deaths.

In this context, we recently reported autoantibodies neutralizing type I interferons (autoAb-IFN) in the first patient to die of H5N1 influenza in the USA.^1^ Another group reported an enrichment of autoAb-IFN in patients infected with H7N9 influenza in China^2^. This led us to search for autoAb-IFN in patients infected by H7N7 in the 2003 Netherlands outbreak^3^. The 89 diagnosed cases included a 57-year-old male veterinary surgeon who died. The other 88 patients had mild conjunctivitis or flu-like illness. We tested serum samples from the only patient to have died and 34 mild cases (aged 13–59 years). We identified autoAbs neutralizing IFN-α, -ω, and -β in the fatal case (Fig. 1A), and autoAbs neutralizing IFN-β in two mild cases (5.7%) aged 30 and 35 years (Fig. 1B). This is unlikely to be due to chance, as autoAbs neutralizing all three subtypes are generally absent in people less than 60 years old, whereas autoAbs neutralizing IFN-β are found in only 0.3% of people of the same age^4^.

Thus, autoAb-IFN may underlie human H7N7, H5N1, and H7N9 infections. The two deaths from H5N1 and H7N7 occurred in persons with neutralizing autoAbs of multiple IFN subtypes. AutoAb-IFN are emerging as major, common, and global determinants of life-threatening disease due to zoonotic viruses (avian influenza), emerging human viruses (SARS-CoV-2 and MERS-CoV), epidemic viruses (seasonal influenza), endemic viruses (West Nile virus, tick-borne encephalitis virus, Usutu virus, Powassan virus, and Ross River virus), and disease due to live-attenuated viral vaccines (yellow fever and Chikungunya vaccines)^4^.

More alarmingly, the genome of the A/Netherlands/219/03 strain isolated from the fatal case with autoAbs reported here, carried mutations causing 14 amino-acid substitutions in viral proteins crucial for adaptation to humans (Table S1)^3^. The genome of the A/Louisiana/12/2024 strain isolated from the fatal case of H5N1 influenza with autoAb-IFN that we reported last year carried similar mutations^1^. Our findings suggest that defective type I IFN immunity enables zoonotic viruses to replicate in human cells, facilitating their adaptation to human host and, therefore increases the possibility of human-to-human transmission^5^. The two mild H7N7 cases testing positive for autoAb-IFN suggest that the breach might occur stealthily. These results and other reports^1,2^ are consistent with persons with autoAb-IFN being at high risk of zoonotic viral infections. We hypothesize that such individuals could potentially act as the “patient zero” in emerging viral pandemics.

## Supplementary Material

Supplementary Appendix

## Figures and Tables

**Figure 1.**
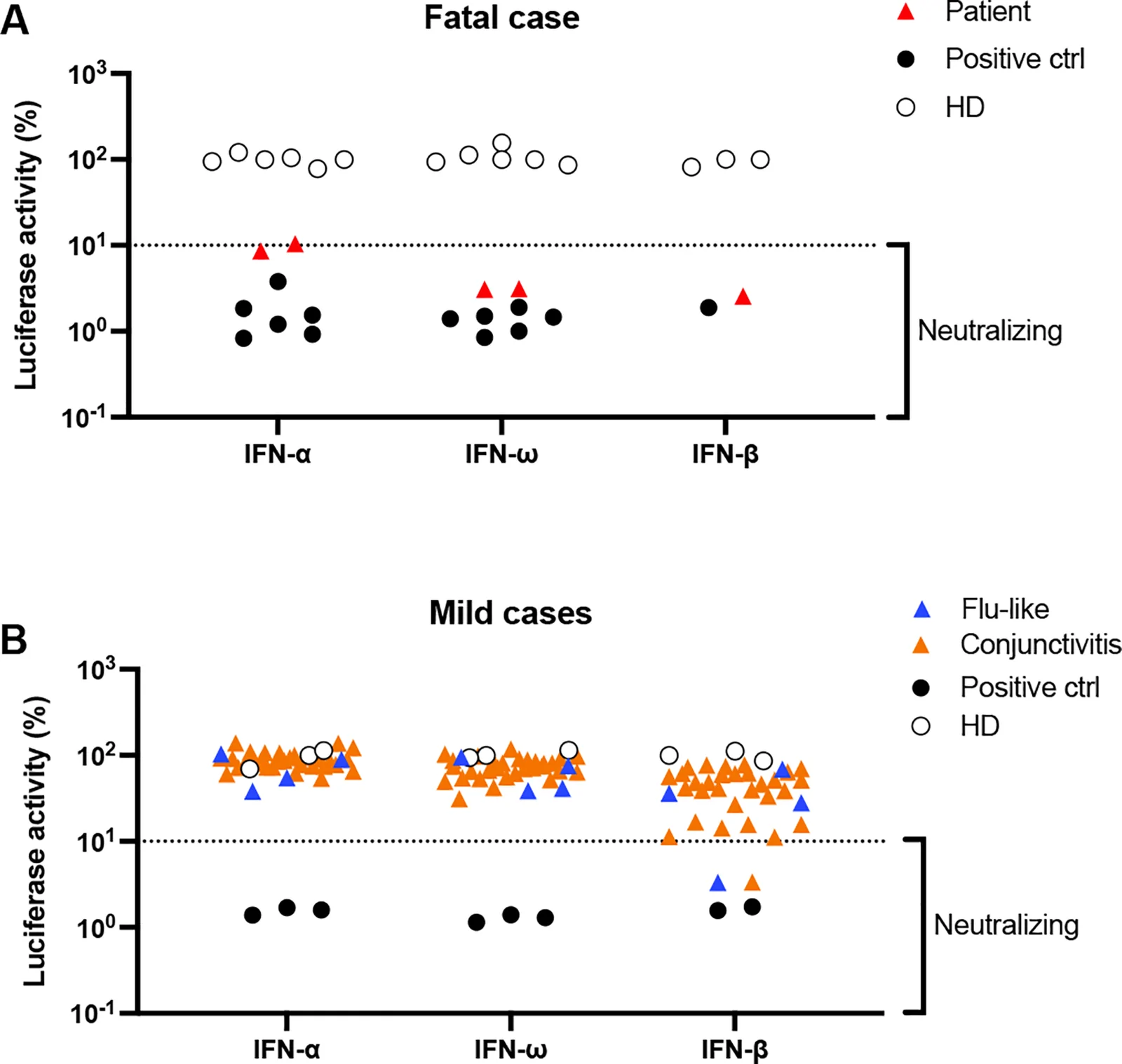
AutoAb-IFN in patients with H7N7 infections. AutoAbs in patient sera neutralized IFN-α, -ω, and -β. A549-IFNreporter (AIR) cells carrying the ISRE reporter were stimulated with IFN-α (100pg/mL), -ω (100pg/mL), or -β (1ng/mL), with or without sera from the fatal case (A), 34 mild cases (B), an autoAbs positive control (positive ctrl) and healthy donors (HD). All samples were diluted 1:20. Renilla luciferase activity was measured 24 h after stimulation. The results are expressed as a percentage of the mean value for HDs. Luciferase activity levels <10% that of HDs were considered to indicate neutralizing activity. Experiments were done twice.
