# Supplementary material for: Autoantibodies Against Type I Interferons and Death from H7N7 Avian Flu
Source: N Engl J Med. Author manuscript; Available in PMC 2026 Nov 12. (PMC13758938; doi:10.1056/NEJMc2608446)
Supplement: Supplementary file 1 [file NIHMS2211697-supplement-Supplementary_Appendix.pdf]

APPENDIX

TABLE S1. VIRAL SUBSTITUTIONS IN A/NL/219/03* 2

Table S1. Viral substitutions in A/NL/219/03*

	Amino acid substitution	Viral gene
1	S79I	PB2
2	V297I	PB2
3	R355K	PB2
4	Q563R	PB2
5	E627K	PB2
6	F666L	PA
7	I13S	HA
8	A143T	HA
9	K416R	HA
10	N308S	NA
11	A346V	NA
12	T442A	NA
13	P458S	NA
14	V137I	NS

*Fouchier RA, Schneeberger PM, Rozendaal FW, et al. Proc Natl Acad Sci U S A 2004;101(5):1356-61.
